# Repeated Cycles of Binge-Like Ethanol Intake in Adolescent Female Rats Induce Motor Function Impairment and Oxidative Damage in Motor Cortex and Liver, but Not in Blood

**DOI:** 10.1155/2018/3467531

**Published:** 2018-09-19

**Authors:** Luanna Melo Pereira Fernandes, Klaylton Sousa Lopes, Luana Nazaré Silva Santana, Enéas Andrade Fontes-Júnior, Carolina Heitmann Mares Azevedo Ribeiro, Márcia Cristina Freitas Silva, Ricardo Sousa de Oliveira Paraense, Maria Elena Crespo-López, Antônio Rafael Quadros Gomes, Rafael Rodrigues Lima, Marta Chagas Monteiro, Cristiane Socorro Ferraz Maia

**Affiliations:** ^1^Laboratory of Pharmacology of Inflammation and Behavior, Faculty of Pharmacy, Institute of Health Science, Federal University of Pará, Belém, PA, Brazil; ^2^Laboratory of Immunology, Pharmacy Faculty, Institute of Health Science, Federal University of Pará, Belém, PA, Brazil; ^3^Laboratory of Ecotoxicology, Nucleus of Tropical Medicine, Federal University of Pará, Belém, PA, Brazil; ^4^Laboratory of Molecular Pharmacology, Institute of Biological Sciences, Federal University of Pará, Belém PA, Brazil; ^5^Laboratory of Microbiology and Immunology of Teaching and Research, Pharmacy Faculty, Institute of Health Science, Federal University of Pará, Belém PA, Brazil; ^6^Laboratory of Functional and Structural Biology, Institute of Biological Sciences, Federal University of Pará, Belém, PA, Brazil

## Abstract

Moderate ethanol consumption (MEC) is increasing among women. Alcohol exposure usually starts in adolescence and tends to continue until adulthood. We aimed to investigate MEC impacts during adolescence until young adulthood of female rats. Adolescent female Wistar rats received distilled water or ethanol (3 g/kg/day), in a 3 days on-4 days off paradigm (binge drinking) for 1 and 4 consecutive weeks. We evaluate liver and brain oxidative damage, peripheral oxidative parameters by SOD, catalase, thiol contents, and MDA, and behavioral motor function by open-field, pole, beam-walking, and rotarod tests. Our results revealed that repeated episodes of binge drinking during adolescence displayed lipid peroxidation in the liver and brain. Surprisingly, such oxidative damage was not detectable on blood. Besides, harmful histological effects were observed in the liver, associated to steatosis and loss of parenchymal architecture. In addition, ethanol intake elicited motor incoordination, bradykinesia, and reduced spontaneous exploratory behavior in female rats.

## 1. Introduction

Ethanol is one of the oldest psychoactive substances and remains the most intoxicating drug widely used by individuals [1]. In addition to having a cultural background and being accepted in almost all organized societies [1, 2], its consumption is favored by its low cost, wide availability, and easy access [2, 3]. However, the abuse of ethanol intake is considered a public health problem with repercussions on the social environment and it causes several clinical complications [4] that range from behavioral alterations and maladaptive long-term consequences to systemic metabolic disruption and liver failure [5].

Actually, our group has been focusing on alcohol versus adolescence versus female gender, through a heavy-drinking protocol [6–10] or a binge-drinking paradigm [11–14]. In this regard, we showed that heavy chronic EtOH intoxication (6.5 g/kg/day during 55 days) during adolescence induced neuronal loss and an expressive reduction in astrocytes in the cerebral cortex of rats [15]. In addition, the chronic EtOH administration may also potentiate the motor impairments and motor cortex damage induced by focal ischemia in female rats [8, 10].

In view of the binge-drinking paradigm, the intermittent ethanol consumption—when high ethanol dose is consumed in a short period of time followed by a period of withdrawal—is the dominant manner of alcohol misuse in adolescents and young adults [16–18]. The National Institute on Alcohol Abuse and Alcoholism (NIAAA) has recommended that this consumption pattern promotes a blood alcohol concentration level of 0.08 g/dL. Such plasmatic blood levels usually occur after the consumption of four or more drinks for women or five or more drinks for men, for a two-hour period [19]. Besides, the frequency of ethanol consumption in a binge manner among adolescents, mainly of university students, occurs 3 days a week [20]. Additionally, this ethanol consumption pattern has increased among the female gender in Brazil, especially with the younger ones [21], indicating that females have become one group that presents higher risks related to the deleterious effects of ethanol; even so, the harmful effects of ethanol among females have been poorly investigated.

In fact, there are sex differences in metabolism and response to binge-like ethanol. Some studies have reported that females display greater susceptibility to acute and long-term alterations of mood and memory after ethanol-intermittent treatment [22–24]. In addition, higher anatomical and histological alterations have been reportedly elicited by binge-drinking consumption in females than in males, which suggest that vulnerability to ethanol damage is gender dependent [25, 26]. Beyond that, oxidative balance also seems to be different between males and females. In an interesting study, Jung and Metzger [27] found an innate difference related to oxidative status displayed by ethanol exposure protocol between males and females, in which hormonal factors may contribute to the possible neuroprotector effect. However, such steroidal protection against oxidative damage was unlikely in the brain.

After ingestion, alcohol is metabolized by three pathways in the liver, that is, alcohol dehydrogenase (ADH), the microsomal ethanol oxidation system (MEOS) by induction of cytochrome P-4502E1 (CYP2E1), and catalase enzymes. These processes result in toxic and highly reactive products such as acetaldehyde and reactive oxygen species (ROS), which makes organs, such as the liver, more susceptible to alcohol-induced damage by different mechanisms that are not clear enough [28, 29]. In the brain, alcohol metabolism also shares all the mechanisms related above, generating acetaldehyde and an excess of H_2_O_2_, which displays oxidative damage as one of the main mechanisms of tissue injury [30, 31].

Actually, alcoholic liver disease (ALD) is among the leading causes of morbidity and mortality worldwide. Their pathophysiology includes a broad spectrum of diseases ranging from simple steatosis to more severe forms of liver injury, such as steatohepatitis, cirrhosis, and hepatocellular carcinoma [32, 33]. Of interest, among the risk factors for ALD, the pattern of alcohol consumption and female display a double risk. In women, the risk is higher due to the deficit of gastric ADH, a higher proportion of body fat, and the presence of estrogen [33].

Allied to metabolic damage, ethanol intake during adolescence has been reported as a neurotoxic drug. The main hazardous effects detected are related to its neurobehavioral alteration, in which motor function is deeply impacted [34]. However, it is still unclear if the exposure of females to ethanol in a binge-like manner causes cumulative effects on motor function from middle to late adolescence, if these effects involve oxidative stress, and how this mechanism reverberates on the motor cortex, liver, and blood. Thus, we now investigated the systemic oxidative stress effects of the binge-like ethanol paradigm and its repercussions on motor function in adolescent female rats.

## 2. Materials and Methods

### 2.1. Animals

Adolescent female *Wistar* rats (*n* = 48; 72.83 ± 0.36 *g*) were obtained from the Animal Facility of the Federal University of Pará (UFPA) and kept in collective cages (four animals per cage). Animals were maintained in a climate-controlled room on a 12:12-h light/dark cycle (lights on 7:00 AM), with food and water *ad libitum.* All procedures were approved by the Ethics Committee on Experimental Animals of the Federal University of Pará under license number BIO-196-14 and followed the guidelines suggested by the National Institutes of Health (NIH) Guide for the Care and Use of Laboratory Animals (2011). Adolescent female rats were used because previous investigations reported that this sex appears to be more resistant to oxidative brain damage ([27], but the ethanol-induced brain injury is more evident in female than in male rodents [25]. Moreover, human female adolescents are more vulnerable than males to the adverse neurodevelopmental effects (i.e., neurotoxic effects in cerebral cortex) of heavy alcohol binge drinking [26].

### 2.2. Experimental Groups and Treatment

**Figure 1:**
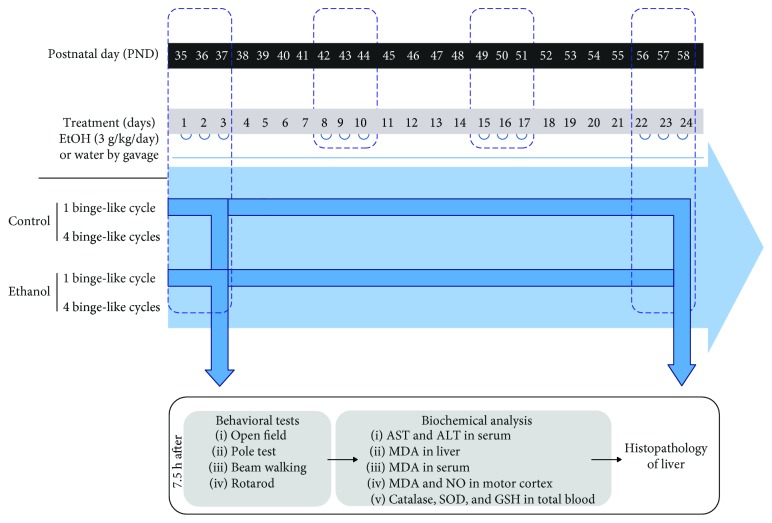
Experimental schedule of binge-like ethanol treatment in adolescent rats. Each cycle of binge-like ethanol administration consisted of a daily gavage administration of ethanol (3 g/kg/day) (or water to control group) for 3 consecutive days weekly. Initially, the immediate postadministration effects of either a single or repeated binge-like ethanol episodes in adolescence were assessed. Female rats underwent one cycle of binge-like ethanol treatment at postnatal day 35 (PND35) or 4 cycles of binge-like episodes (PND35–58). After motor behavioral analysis, rats were sacrificed for markers of biochemical analysis and hepatic histopathology.

In this study, one episode of binge-like ethanol treatment was considered equivalent to a single daily intragastric administration of ethanol (3.0 g/kg/day, 20% w/v ethanol) for three consecutive days [35] to mimic the pattern occurring in human adolescents [20]. On postnatal day (PND) 35, all rats were randomly assigned to either (i) ethanol or (ii) water control groups at two different periods. One group of animals (PND 35–37) received 1 cycle of binge-like treatment (acute binge ethanol-treated adolescent rats). Other animals received 4 weeks of treatment (4 binge-like ethanol cycles), from PND 35–58, which mimics from middle to late adolescence (for review see [36]). See Figure 1 for the timeline of the experimental design.

All animals received a single daily intragastric administration of ethanol (dose of 3.0 g/kg/day, 20% w/v ethanol) on 3 consecutive days on schedule—always between 7:00 AM and 8:00 AM [35]—and 4 consecutive days off schedule. Control subjects received comparable volumes of distilled water, according to the procedure previously described [37]. The animals' weight gain was measured prior the beginning of ethanol treatment and weekly. Moreover, the survival rate of animals was assessed throughout the entire experimental protocol period and no death was observed within repeated intermittent ethanol administration.

### 2.3. Behavioral Tasks

Seven and a half hours after the period of binge-like ethanol treatment, animals were conducted to the test room for acclimatization and habituation to the test environment for one hour. The behavioral tests (open-field, pole, beam-walking, and rotarod tests) were employed between 11:00 AM and 6:00 PM in a sound-attenuated room under low-intensity light (12 lux).

#### 2.3.1. Open Field

We used an open-field apparatus to evaluate spontaneous exploratory activity to assess locomotion [38]. The rats were placed individually at the center of the open-field arena (100 × 100 × 40 cm) and were permitted to allow spontaneous locomotor activity in the apparatus for 5 min. To evaluate the horizontal and vertical locomotor activities, the total distance traveled and number of rearings were measured, as previously described [39]. The rat's activity was video monitored by a camera positioned above the arena to be analyzed off-line with the ANY-maze™ (Stoelting, USA) software by two observers blind to the treatments. The rearing parameter was manually counted.

#### 2.3.2. Pole Test

The pole test, initially described by Ogawa et al. [40], is an experiment used to evaluate movement disorders, especially bradykinesia, characterized by an increase in latency for the execution of movements [41]. In the test, the animal is confronted with the situation of turning the body and descending from a vertical beam [38]. The equipment comprises a rough vertical beam (2 × 50 cm) supported on a circular platform (1 cm height; *r* = 25 *cm*). The task consists of the subject's ability to turn haed down and descend to the safe platform. Briefly, animals were placed head upward on the top of the beam to perform the task in five attempts, at intervals of 60 s, in which escape latency was registered (cutoff 120 s), according to da Silva et al.'s [6] protocol. The three best scores were considered for each rat. Animals unable to conclude the task were assigned the maximum time.

#### 2.3.3. Beam-Walking Test

The beam-walking test is used to assess motor strength and balance [42]. In fact, Carter et al. [43] suggested that this test is useful tool to motor coordination and refinement analysis. The wood apparatus consists of suspended beams (1 m) elevated 50 cm from the floor [43]. The serial beams presented 2 types of cross sections: square (28, 12, and 5 mm) and circular (28, 17, and 11 mm), both of which are linked to a secure box [38]. Initially, animals were habituated on the squared 28 mm beam for 120 s. After that, animals were submitted to each beam (from square to round) in a decreased cross-sectional area for 60 s with an interval of 60 s. Motor coordination and balance in spontaneous activity were assessed through the number of slips during the test section of each beam (adapted from [44]).

#### 2.3.4. Rotarod

The rotarod test is widely used to assess motor coordination, balance, and ataxia [38]. Usually, it is used as a motor performance evaluation test on a rotarod apparatus (Insight®, Brazil) because it is based on measuring the ability of rodents to maintain balance on a rotating cylinder driven by a motor. The apparatus consists of a grooved metal roller (8 cm in diameter), separated into 9 cm-wide compartments elevated 16 cm. In the test training, the animals were placed on the spin axis for a period of 120 seconds at 8 rpm. After the training, the test was performed in three successive exposures of 120 seconds at 8 rpm with an intertrial interval of 60 s [6, 15]. The parameter evaluated was the latency to first fall.

### 2.4. Biochemical Assays

#### 2.4.1. Samples

After behavioral assays, animals were euthanized by cervical dislocation for biochemical evaluations and histopathological note. The blood content was obtained by heart puncture and collected in tubes; concomitantly, livers and brains were removed and cooled on dry ice. Then, the motor cortex tissue was dissected from the brain and both of the tissues were frozen in liquid nitrogen. For analysis, tissue samples were thawed and resuspended in 20 mM Tris-hydrochloride (Tris-HCl) buffer, pH 7.4, at 4°C for sonic disintegration. Results were expressed as percentages of control groups. The blood, liver, and motor cortex were stored at −80°C for determination of the biochemical markers of damage. The serum was obtained by centrifugation for 10 min at 1400 ×g and stored at −80°C.

#### 2.4.2. Biochemistry Parameter Analysis

Serum activities of aspartate and alanine aminotransferase (AST and ALT, respectively) were measured by the veterinary laboratory device Reflotron Plus (Roche Diagnostics).

#### 2.4.3. Oxidative Biochemistry in Blood

*(1) Determination of Malondialdehyde (MDA)*. Determination of malondialdehyde (MDA) is a method that evaluates lipid peroxidation and acts as an indicator of oxidative stress. The method is based on the reaction of MDA, among other substances, with the reaction of thiobarbituric acid reactive substances (TBARS) performed according to a previously [45] proposed method [46]. Onto each test tube, 10 nM of TBA (Sigma-Aldrich T5500) and 0.5 mL of the sample serum were added. Then, the tubes were placed in a water bath at pH 2.5 and at a high temperature (94°C × 60 min) to form the pink-coloured MDA-TBA complex. After this procedure, the samples were cooled in running water and then butyl alcohol was added to each sample to obtain the maximum extraction of MDA into the organic phase. Finally, the tubes were centrifuged at 2500 rpm for 10 minutes and the supernatant was collected and read by the spectrophotometric method (wavelength of 535 nm). Results were expressed as percentages of control groups.

*(2) Superoxide Dismutase (SOD) Activity*. Determination of superoxide dismutase (SOD) activity was performed according to the procedure recommended by McCord and Fridovich [47]. For this, blood samples were haemolysed into ice water (1 : 3) and then diluted in a Tris-based buffer (Tris 1 M/EDTA 5 mM, pH 8.0). This method evaluated the ability of SOD to catalyse the conversion of O^2−^ to H_2_O_2_ and O_2_. SOD activity was measured using UV spectrophotometry at a wavelength of 550 nm.

*(3) Catalase Activity*. Catalase activity was determined by measuring the rate of enzymatic decomposition of H_2_O_2_ (10 mM) to H_2_O and O_2_. Blood samples were haemolysed into ice water (1 : 3) and then diluted in a Tris-based buffer (Tris 1 M/EDTA 5 mM, pH 8.0). The decay of H_2_O_2_ was measured using ultraviolet spectrophotometry at 240 nm, and enzyme activity was expressed in CAT units, where one unit is the amount of enzyme needed to hydrolyze 1 *μ*mol of H_2_O_2_/min/mg protein.

*(4) Content of Thiol Groups*. The content of thiol groups was based on the ability to reduce 5,5-dithiobis-2-nitrobenzoic acid (DTNB) for nitrobenzoic acid (TNB), wherein haemolysed blood samples (20 *μ*L) were solubilized with distilled water plus PBS/EDTA (4 mL) and mixed by vortexing according to Belém-Filho et al.'s [11] protocol. After this, 3 mL of samples was quantitated by spectrophotometry at 412 nm and the content of the thiol groups were expressed in *μ*g/mL, as described by Riddles et al. [48].

#### 2.4.4. Oxidative Biochemistry in Tissue Samples

*(1) Lipid Peroxidation Levels in Liver and Motor Cortex*. Levels of lipid peroxidation in the liver and motor cortex samples was evaluated by a method based on the reaction between MDA and N-methyl-2-phenylindole [49], expressed as moles of MDA per milligram of protein. An aliquot of homogenate was centrifuged at 2500 ×g for 30 min at 4°C, and the supernatant was used for the reaction with N-methyl-phenyl indole (NMFI) and methane sulfonic acid at 45°C, during 40 min, yielding a stable chromophore measured at 570 nm wavelength and compared with the standard curve of MDA and corrected with the protein concentration of each sample [8, 12].

*(2) Determination of Nitrate/Nitrite (NOx).* According to Griess method [50], NOx was measured. In short, an aliquot of cerebral cortex homogenate was centrifuged at 21,000 rpm for 10 minutes at 4°C, and the supernatant was used to analyze nitrite levels. After this, fifty microliters of the supernatant sample or standard sodium nitrite solution was mixed with another 50 *μ*L of the Griess reagent (0.1% N-(1-naphthyl) ethylenediamine dihydrochloride; and 1% sulfanilamide in 5% phosphoric acid; 1 : 1) and maintained at room temperature for 20 minutes. Subsequently, the tissue samples were analyzed on a spectrophotometer by absorbance at 550 nm and compared to that of standard solutions of sodium nitrite. The standard curve was elaborated by sodium nitrate (NaNO_2_) which reflected NOx concentration and data were corrected with the protein concentration of each sample.

All results above were expressed as percentages of the control groups [8].

*(3) Determination of Protein Content*. Total protein content in the supernatants was assayed using the Bradford [51] methodology. Hepatic and cortical protein oxidation was measured wherein an aliquot of homogenate was incubated with the Bradford reagent (5% ethanol; 8.5% phosphoric acid; 0.25% Coomassie Brilliant Blue G-250) for 5 min at room temperature. The absorbance was measured at 570 nm and compared to standard solutions of bovine serum albumin. Results were used for the correction of MDA and nitrite concentrations [8, 12].

### 2.5. Histopathology of the Liver

After removing the liver, liver tissues were fixed in 10% buffered formalin, embedded in paraffin, sectioned (5 *μ*m thick), and stained with hematoxylin and eosin (HE). All sections were stained and surveyed on a light microscope (Nikon Eclipse E200). Illustrative images from all experimental groups were obtained using a digital camera attached to the microscope (Nikon Eclipse 50i), using the software Moticam 2500 for qualitative analysis.

### 2.6. Statistical Analysis

Values are expressed as mean ± S.E.M. of *n* = 9–12 animals per group for motor behavior and *n* = 3–6 per group for biochemical analysis. Statistical comparison was performed by two-way ANOVA for treatment (water *vs*. ethanol) and after different binge-like ethanol cycles (after 1 cycle *vs*. after 4 cycles) as variables; multiple post hoc comparisons were performed using the Fisher-LSD test and *P* < 0.05. Rotarod and beam-walking tests were analyzed by two-way ANOVA with repeated measures (successive sessions) followed by the Fisher-LSD post hoc test.

## 3. Results

### 3.1. Impact of Repeated Cycles of Binge-Like Ethanol Exposure Causes Worse Motor Impairment in Female Adolescent Rats

**Figure 2:**
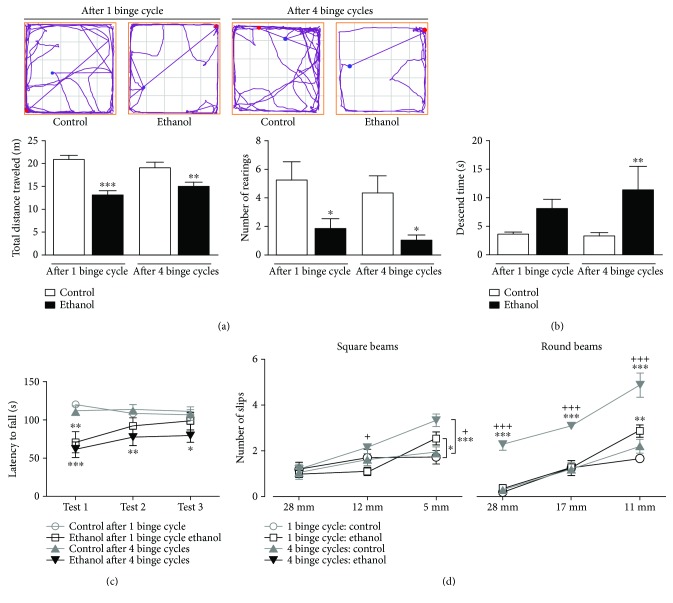
Impact of adolescent binge-ethanol exposure on motor behavior of female rats. The analysis of the total distance traveled accessed the horizontal locomotor activity, whereas the number of rearing informed the vertical locomotor activity, both measures being derived from the track plots in the open-field arena (a). (b) The descent time from vertical beam to the platform base at the pole test is shown as a measure of the kinetics of movement. (c) The latency to first fall on the cylinder of the rotarod apparatus is illustrated as a measure of the motor coordination and balance by forced locomotor activity in the rotarod test. (d) The number of slips to cross the series of graduated beams (square and round) is shown to access the motor learning, coordination, and balance by spontaneous locomotor activity in the beam-walking test. Data are mean ± SEM of *n* = 9–12 rats per group. ^∗^*P* < 0.05, ^∗∗^*P* < 0.001, and ^∗∗∗^*P* < 0.0001*vs*. age-matched control; ^+^*P* < 0.05 and ^+++^*P* < 0.001*vs*. ethanol-treated after 1 binge-like cycle, all assessed using a Fisher-LSD test after two-way ANOVA.

The spontaneous locomotor activity, assessed by horizontal and vertical exploration (total distance traveled and number of rearing parameters, respectively) on the open-field test, is demonstrated in Figure 2(a). Two-way ANOVA (ethanol-treatment *vs.* repeated cycles) of motor behavior, assessed by horizontal and vertical exploration, revealed a significant difference only for effects of binge-like ethanol treatment (*F*(1, 44) = 33.485; *P* = 0.001 and *F*(1, 37) = 11.826; *P* = 0.001, respectively). Fisher-LSD post hoc comparisons showed that binge-like ethanol treatment induces reduction of the horizontal and vertical exploratory activity of adolescent female rats after 1 (*P* < 0.001; *P* = 0.017, respectively) and 4 binge cycles (*P* = 0.007; *P* = 0.023, respectively) of ethanol treatment.

In the pole test (Figure 2(b)), two-way ANOVA of the descent time from the vertical beam to the platform base showed a significant difference for the effect of binge-ethanol treatment (*F*(1, 39) = 6.225; *P* = 0.017). Fisher-LSD post hoc comparisons revealed that after 4 repeated cycles of binge-ethanol exposure, adolescent rats displayed an increased latency to descend from the beam to the platform in the pole test (*P* = 0.028).

To evaluate the repercussions of repeated cycles of binge-like ethanol on motor learning, coordination, and balance in adolescent rats, we subjected the animals to three consecutive sessions (8 rpm) on the rotarod apparatus (Figure 2(c)). Two-way ANOVA with repeated measures revealed a significant difference at latency to first fall of the cylinder of the rotarod apparatus for effects of binge-like ethanol treatment (*F*(3, 47) = 10.858; *P* = 0.001). Fisher-LSD post hoc comparisons revealed that a single binge-like cycle in adolescence provokes impairment on motor coordination and balance in female rats at first presentation to the rotarod apparatus (*P* = 0.001) that was recovered on the subsequent sections of the test. However, repeated binge-ethanol cycles during adolescence reduced the animal's dwell time on the rotarod's scrollbar in all successive sessions (first, second, and third) when compared to their counterparts (*P* = 0.0001, *P* = 0.033, and *P* = 0.047, respectively).

Figure 2(d) represents the performance of ethanol-treated adolescent female rats on the square and round beams of the beam-walking test. Two-way ANOVA with repeated measures revealed a significant difference at the number of slips to cross the suspended square beam by binge-like ethanol treatment (*F*(3, 52) = 3.261; *P* = 0.03), as well as related to the thickness of the cross-sectional area beams (*F*(2, 52) = 26.942; *P* = 0.0001). Fisher-LSD post hoc comparisons revealed that a single binge-like exposure generates an increase in the number of slips on the squared thinner beam (5 mm; *P* = 0.044). Indeed, repeated cycles of adolescent binge-like ethanol significantly increased the number of slips during this section of the beam, except for the larger beam (28 mm; intermediary: *P* = 0.012; and thinner cross-sectional area: *P* = 0.0001, respectively).

In order to make the task more complex, as well as to evaluate motor learning, following square beam sessions, animals were submitted to the round beams (Figure 2(d)). Two-way ANOVA with repeated measures revealed a significant difference in the number of slips to cross the suspended round beam of binge-like ethanol treatment (*F*(3, 61) = 48.444; *P* = 0.0001), as well as the thickness of the cross-sectional area beams (*F*(2, 61) = 48.955; *P* = 0.0001). Fisher-LSD post hoc comparisons revealed that a single binge-like exposure increased the number of slips on the thinner beam (11 mm; *P* = 0.009). However, the number of slips per session was significantly increased after repeated cycles of adolescent binge-like ethanol in all diameters evaluated (*P* = 0.0001 for all thickness of beams).

### 3.2. Repeated Cycles of Binge-Like Ethanol Treatment Elicit Accumulative Effects on Hepatic Damage in Adolescent Rats

**Figure 3:**
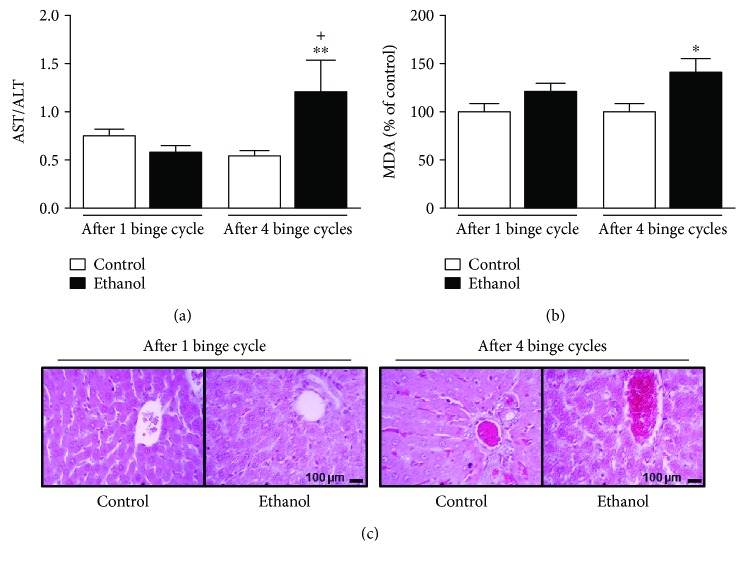
Postconsumption effects of repeated binge-like ethanol treatment on hepatic function of adolescent female rats. The analysis of the ratio of serum activities of aspartate aminotransferase (AST) on alanine aminotransferase (ALT) (a) and percental of malondialdehyde (MDA) levels in hepatic tissue (b). (c) The histopathology of hepatic tissue analyzed by hematoxylin/eosin (HE) staining as morphological qualitative evaluation is shown. Data are mean ± SEM of *n* = 3–6 animals per group. ^∗^*P* < 0.05 and ^∗∗^*P* < 0.001*vs*. age-matched control; ^+^*P* < 0.05*vs*. ethanol-treated after 1 binge-like cycle, all assessed using a Fisher-LSD test after two-way ANOVA. Scale = 100 *μ*m.

Two-way ANOVA (ethanol-treatment *vs.* repeated cycles) of liver damage, assessed by the ratio of serum activity of hepatic transaminases (AST/ALT) and tissue malondialdehyde (MDA) levels (Figures 3(a) and 3(b)), revealed a significant difference for the interaction between effects of binge-like ethanol treatment and repeated cycles (*F*(1, 16) = 5.807; *P* = 0.028) for the ratio AST/ALT, but not for MDA levels that showed only significance for effects of binge-like ethanol treatment (*F*(1, 12) = 5.671; *P* = 0.035). Fisher-LSD post hoc comparisons confirmed hepatocyte oxidative damage by high levels of MDA (*P* = 0.045) (Figure 3(b)) and hepatic transaminases (*P* = 0.016) (Figure 3(a)) in adolescent female rats after 4 cycles of binge-like ethanol treatment.

The histopathological study revealed that control group animals presented normal aspects of the cellular, extracellular, and vascular components of the liver in both periods studied in this experiment. In the ethanol-treated groups, in the acute period corresponding to the 1 binge-like ethanol cycle, the following was observed: intense microsteidosis, alteration in the morphology of the hepatocytes, and tissue parenchyma on the centrilobular region that extended to the mid-zonal region, being more scarce or having more focal segments in the peripheral and superficial segments. In the group submitted to 4 binge-like ethanol cycles, an intense microsteidosis associated to vascular congestion and visible loss of the structural cohesion of the parenchyma were observed (Figure 3(c)).

### 3.3. Adolescent Binge-Like Ethanol Treatment Induces Accumulative Effects on Oxidative Damage in Motor Cortex, Not Detectable Peripherally

**Figure 4:**
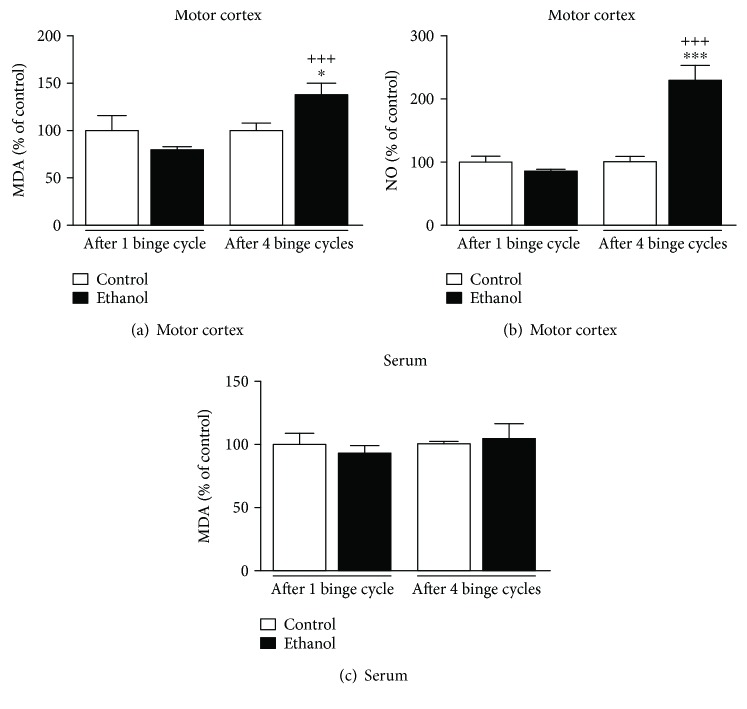
Binge-like ethanol administration on the marker of oxidative damage in the motor cortex and serum of adolescent female rats. The percental of malondialdehyde (MDA) levels in the motor cortex (a) and serum (c) of female rats. The percental of nitrite (NO) levels in the motor cortex (b). The results are mean ± SEM of *n* = 3–6 animals per group. ^∗^*P* < 0.05 and ^∗∗∗^*P* < 0.0001*vs*. age-matched control; ^+++^*P* < 0.001*vs*. ethanol-treated after 1 binge-like cycle, all assessed using a Fisher-LSD test after two-way ANOVA.

Two-way ANOVA (ethanol-treatment *vs.* repeated cycles) of motor cortex damage, assessed by the tissue malondialdehyde (MDA) levels (Figure 4(a)), revealed significant effects of repeated cycles (*F*(1, 12) = 9.159; *P* = 0.011) and their interaction with the effects of binge-like ethanol treatment (*F*(1, 12) = 9.159; *P* = 0.011). Fisher-LSD post hoc comparisons confirmed oxidative damage in the motor cortex by high levels of MDA (*P* = 0.022) in adolescent female rats after 4 cycles of binge-like ethanol treatment. In addition, our results showed an increase of NO production in the 4 binge-like ethanol cycle animals compared to control subjects (*P* < 0.0001; Figure 4(b)). In contrast, the levels of the lipid peroxidation marker in the serum did not indicate peripheral oxidative imbalance, even after 4 cycles of ethanol treatment (Figure 4(c)).

### 3.4. Repeated Cycles of Binge-Like Ethanol Administration during Adolescence Cause Change in Enzymatic and Nonenzymatic Oxidative Response in Adolescent Female Rats

**Figure 5:**
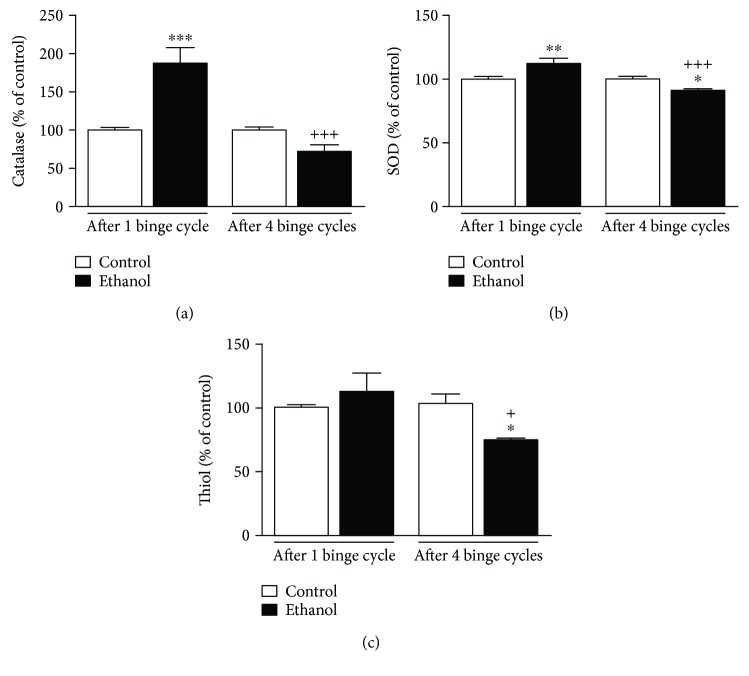
Effects of adolescent binge-ethanol paradigm on the oxidative enzymatic (catalase and superoxide dismutase (SOD)) and nonenzymatic (thiol groups) activities in blood of female rats. Oxidative biochemical of catalase (a) and SOD activities (b) after binge-like ethanol administration, both illustrated as % of control. (c) The content of thiol (% of control) after binge-like ethanol administration in adolescent female rats is shown. Results are mean ± SEM of *n* = 4 − 5 rats per group. ^∗^*P* < 0.05, ^∗∗^*P* < 0.001, and ^∗∗∗^*P* < 0.0001*vs*. age-matched control; ^++^*P* < 0.01 and ^+++^*P* < 0.001*vs*. ethanol-treated after 1 binge-like cycle, all assessed using a Fisher-LSD test after two-way ANOVA.

Two-way ANOVA (ethanol-treatment *vs.* repeated cycles) of peripheral antioxidant balance was assessed by the levels of enzymatic and nonenzymatic antioxidant agents (Figures 5(a) and 5(c)). Effects of binge-like ethanol treatment revealed a significant effect for catalase (CAT) (*F*(1, 10) = 6.446; *P* = 0.029). In addition, the repeated cycles significantly influenced CAT (*F*(1, 10) = 26.397; *P* = 0.001) and SOD (*F*(1, 14) = 17.286; *P* = 0.001). On the other hand, CAT (*F*(1, 10) = 26.397; *P* = 0.001), SOD (*F*(1, 14) = 17.286; *P* = 0.001), and content of thiol (*F*(1, 15) = 5.195; *P* = 0.038) were significantly induced by the interaction of these factors. Fisher-LSD post hoc comparisons showed a significant increase in CAT (*P* < 0.001) and SOD (*P* = 0.006) levels in adolescent female rats after 1 cycle, with successive depletion of these enzymes on the 4th cycle of binge-like ethanol treatment as well as reduction in the formation of thiol groups (*P* = 0.045).

## 4. Discussion

The present study evidenced that the binge-like pattern of ethanol intake during adolescence in female rats induces marked liver and motor cortex oxidative stress related to repercussions on motor function. More importantly, such alterations were not reflected on the peripheral oxidative damage.

Lipid peroxidation (LPO) is a natural degenerative process in which there are interactions involving ROS and polyunsaturated fatty acids of biological membranes, jeopardizing the integrity of organelles and the cell itself [52, 53]. Alcohol consumption intensifies such a process due to ROS production during its metabolism and selectively alters mitochondrial function of the liver and other tissues [54–56]. Our data revealed that binge drinking during adolescence altered hepatic health. In fact, a single acute dose of ethanol was not sufficient to display liver alteration. However, after 4 cycles of ethanol exposure, the AST/ALT as well as the MDA levels were increased compared to its counterparts, showing liver injury caused by the ingestion of ethanol. In addition, the first episode of binge drinking resulted in microvesicular and macrovesicular steatosis in zone 3 (perivenular) that constitutes the first response of the liver to alcohol abuse. Such initial hepatic alteration was intensified by the subsequent ethanol exposure that aggravated the initial damage and reached other tissue components, thus causing vascular congestion and loss of the structural cohesion of the parenchyma. In fact, there is a consensus that alcohol is a hepatotoxic drug that also disrupts lipid metabolism. Alcohol inhibits the mitochondrial *β*-oxidation of fatty acids of hepatocytes and induces increased mobilization of fatty acids from adipose tissue to the liver, increasing fat accumulation [57]. However, it is the first time that it was demonstrated that a single binge-drinking episode during adolescence alters the hepatic tissue in females. In addition, we showed that the consumption of ethanol, even followed by withdrawal periods (4 days off), from adolescence until young adulthood aggravated the previous damage. Recently, Choi et al. [58] showed that chronic binge exposure led to ROS level overproduction and elevated amounts of the microsomal ethanol-oxidizing enzyme (i.e., CYP2E1 and NADPH oxidase), but not of iNOS, and decreased cell antioxidant defense (i.e., GSH and mitochondrial ALDH2 activity) in the liver.

Allied to the liver, our data also showed that after consumption of chronic binge drinking (after 4 binge cycles) in early adolescence, the antioxidant enzyme system of both CAT and SOD activities were downregulated. The reduced defense against oxidative damage may particularly affect the organs more susceptible to this type of damage (i.e., the liver and brain), becoming a major molecular contribution for the deleterious consequences observed in behavioral alterations. After this initial approach for the evaluation of the oxidative status carried in our work with CAT and SOD and based on our data, a detailed study of the detoxification enzyme systems, including glutathione peroxidase, iNOS, and NADPH oxidase, could contribute to a better understanding of the molecular processes involved in the early consequences of ethanol consumption.

Brain cells are more vulnerable to oxidative damage related to reduced levels of antioxidant enzymes and a high level of oxidant metabolism [59]. In this regard, our previous studies had already shown that the chronic EtOH exposure during adolescence reduced the astrocyte population and the number of neurons and glia cells with an increase in both nitric oxide and lipid peroxidation in the cerebral cortex [15].

EtOH is known to induce oxidative imbalance by increasing the ROS products generated by its metabolism, causing the reduction of enzymatic antioxidants, and increasing the biomarkers of macromolecules [28, 60, 61]. The main route of the alcohol metabolism is performed by the hepatic enzyme system; however, other routes can be used, such as the oxidation by the CAT antioxidant enzyme, which acts as a limited-step pathway [62]. Thus, increased systemic oxidative stress and MDA levels, as well as activation of neuroapoptotic and neuroinflammatory pathways, have also been previously associated with alcohol abuse [63, 64]. Thus, this enhanced oxidative stress may systemically contribute to cellular damage, as well as damage in the brain, leading to neurodegeneration, development of cognitive impairment, anxiety, depressive like behavior, and other psychiatric disorders [65, 66]. These reactions also promoted a reduction in CAT and SOD activity and thiol (-SH) group levels peripherally (Figures 5(a)–5(c)), possibly reflecting on the motor cortex, leading to an increase in NO and MDA levels in the motor cortex (Figures 4(a) and 4(b)). In this sense, functional motor impairment appears as a result, since such area plays a central role in both motor skill learning and execution [67]. In fact, the relationship between motor function and oxidative disturbance has been reported by other studies (for a review, see [30, 31]).

Although motor function impairment also depends on the period and time of consumption of ethanol, it is known that adolescents are less vulnerable to motor damage compared to adults because younger people are more resistant to the drug's sedative effects [68, 69]. This characteristic of adolescence can lead to increased consumption of large amounts of alcohol without generating the perception of toxicological effects.

The exploratory tendency of the animal in a new environment in the open-field test can be translated as spontaneous locomotor activity, using the parameters of total distance traveled in the arena as a measure of horizontal exploration and frequency of rearings resulting from vertical exploration [38, 39]. This study demonstrated that horizontal and vertical exploratory activities were impaired after administration of ethanol in the binge pattern. Pascual et al. [70] evaluated the effect of chronic voluntary consumption of EtOH (mean 10 g/kg) initiated at the end of the periadolescence (42–49 PND) of rodents during 5 months of intoxication. These authors found a reduction in the spontaneous locomotor activity of the animals within 8 hours after the removal of the EtOH solution, but this loss did not last for a prolonged period (15 days). In contrast, our study demonstrates that the intermittent and episodic administration of ethanol (3 g/kg) from adolescence to adulthood reduces the exploratory locomotor activity of the animals in the early withdrawal. These findings indicate the greatest harmful effect of alcohol consumption on the usual binge-drinking model among adolescents.

In order to cover the study on the various aspects of motor function, the behavioral tests of the pole test, beam-walking, and rotarod assays were used. The first one was developed to measure bradykinesia and reduction of muscle tone, evaluating the animal's ability to invert its axis and descend to the platform [38, 71]. On the other hand, the latter two evaluated coordination and motor balance [38, 42]. The development of bradykinesia is measured by the prolongation of the turnaround and descent time of the animal at the platform base [72]. This symptom is associated with abnormal functioning of the intrinsic circuitry of the basal nuclei through the selective hypoactivity of the supplemental motor area and the cortical areas, which are frontal association areas that receive subcortical entry mainly from the basal ganglia [73, 74].

Accordingly, the EtOH-intoxicated groups presented higher scores to perform the test, indicating that the EtOH intoxication, in the way of binge drinking, generated a kinetics loss of the motor activity of the animals. Thus, our findings indicate impairment in subcortical and/or subcortical connection with cortical motor areas in adolescents after repeated binge-like cycles in adolescence.

The rotarod test measures forced gait disturbances and gross motor coordination by an animal's ability to remain on a rotating rod [38]. In the present study, the administration of ethanol in the binge pattern elicited poor coordination on the first section of the test. However, the individual rats increased their skills for remaining on the rotating rod in the subsequent sections, except for animals exposed to ethanol during the adolescence until adulthood (4 cycles of ethanol). In addition to the rotarod apparatus, we employed the beam-walking paradigm to detect fine coordination through a spontaneous motor task [75]. According to the beam-walking test, motor impairment related to ataxic and dystonic characteristics can be assessed through the animal's difficulty in crossing beams of different shapes and cross-sectional areas [38]. Our study demonstrated that the administration of ethanol in the binge-drinking model elicited poor performance on the test, increasing the number of slips, mainly after 4 binge cycles. Such impairment was intensified proportionally to the difficulty of the test sections.

In this sense, for movement to occur correctly, motor areas related to the organization and control of movement (e.g., primary motor cortex, supplemental motor area, premotor, and cingulate motor) influence descending pathways and medullary interneurons through their connections with the brainstem, basal nuclei, and cerebellum [76, 77]. Thus, ataxias result from disorders of the cerebellum and/or its connections, as well as the circuitry that integrates the motor cortex and striatum [78]. Motor incoordination is often used as an index of intoxication produced by drugs that depress the central nervous system [79]. It has been observed that EtOH in the abstinence period can downregulate gabaergic neurons associated with exacerbation of the glutamatergic pathway in most areas of the brain, promoting behavioral effects typical of this period as altered motor control, learning, and memory [31, 80, 81]. Thus, the results obtained in this study reveal that repeated episodes of binge drinking increased the number of slips on the beam-walking test, as well as reduced rotarod latency probably due to these molecular events that may occur in the abstinence period. Thus, we propose that despite the intermittent periods of ethanol withdrawal, the binge-drinking pattern displays liver and motor cortex oxidative damage, which reflects on motor function in female rats. Surprisingly, such tissue damage was not detectable in blood.

Due to the deleterious effects of alcohol abuse and alcoholism, clinical studies aim at characterizing the early events leading to alcoholic diseases (i.e., motor impairment and liver damage) in order to define biomarkers, which may help clinicians establish preventive measures [82]. However, few studies have been proposed to evaluate biomarkers of early detection that might be present in individuals known as “social drinkers” or binge drinkers [83]. Muñiz-Hernández et al. [83] highlighted in their review that biomarkers that reflect oxidative damage—mainly MDA—could be used to identify damages in the earlier stages of ethanol consumption. However, for the first time, our experimental data contradict such hypothesis and reveal that MDA levels are not well indicated for early damage during the adolescent period. Particularly, we suggest that in clinical practice, blood MDA levels could not be used as a peripheral marker for ethanol misuse, since such biomarker in blood is nonspecific. Actually, we highlight that 4 binges do not cause significant lipid peroxidation in the blood despite the presence of high levels of MDA in hepatic and motor cortex tissues associated to motor impairment and hepatic steatosis.

## 5. Conclusions

It is noteworthy that the binge-drinking exposure during adolescence in females may impact liver homeostasis. Besides, the liver and motor cortex seem to be more vulnerable to oxidative damage, even when systemic oxidative damage was not observed. Besides, our results show that motor alteration was altered by a binge-like ethanol paradigm, even after only one exposure. However, bradykinesia, poor coordination, and balance were impaired by subsequent administration of ethanol during adolescence until early adulthood. Would lipid peroxidation be an adequate biomarker for the hazardous effects of ethanol misuse?

## Data Availability

The behavioral and oxidative data used to support the findings of this study are available from the corresponding author upon request.
